# The influence of methamphetamine utilization patterns and adverse childhood experiences on Methamphetamine Use Disorder and Methamphetamine-Induced Psychosis

**DOI:** 10.1192/j.eurpsy.2025.315

**Published:** 2025-08-26

**Authors:** N. A. Tran, R. Kalayasiri

**Affiliations:** 1 Department of Psychiatry, Chulalongkorn University, Bangkok, Thailand

## Abstract

**Introduction:**

Methamphetamine (MA) is one of the most addictive drugs globally. Among its harmful consequences, methamphetamine use disorder (MUD) and methamphetamine-induced psychosis (MAP) are prevalent and increase the burden of mental health worldwide. Recent studies highlighted the relationship of the disorders and various factors including patterns of MA consumption and adverse childhood experiences (ACEs). Understanding the association between these factors and MUD and MAP is essential for advancing our knowledge and improving healthcare for our patients.

**Objectives:**

To investigate the association of MA use patterns, ACEs and the development of MUD and MAP.

**Methods:**

This study analyzed data from a survey using the ThaiMIND questionnaire (September 2023 – June 2024). We collected participants’ socio-demographic details (including gender, age, income, employment, marital status, education), mental health history, other substances use, MA use patterns, ACEs, psychotic symptoms and their onset. The diagnosis of MUD and MAP were based on DSM-5 criteria. Univariate logistic regression was employed to examine the relationships, adjusting for socio-demographics and mental health history for MUD models, and adding other substances use and MUD diagnosis for MAP models.

**Results:**

In this study of 2,524 participants, 1,987 (78.72%) met the criteria for MUD, and 876 (34.71%) met the criteria for MAP. The use of yaba (MA or speed pill) reduced the risk of MAP compared to ice (crystalline MA)(OR = 0.32 [0.12 – 0.85]) while combining two types of MA raised the risk of MAP compared to ice alone (OR = 1.96 [1.37 – 2.81]). For MUD, more frequent MA use, compared to monthly or less, increased the risk with OR = 1.81 [1.34 – 2.43] (2-4 times/month), 2.27 [1.58 – 3.27] (2-3 times/week), and 4.00 [2.87 – 5.59] (4 or more times/week). Similarly, for MAP, using MA 2-3 times/week raised the risk (OR = 1.59 [1.14 – 2.22]), and using it more than 4 times/week further increased the risk (OR = 2.16 [1.62 – 2.87]). Additionally, MA injection significantly heightened the risk of MUD (OR = 6.29 [3.28 – 12.04]). Emotional abuse (OR = 1.84 [1.36 – 2.47]) and physical abuse (OR = 1.71 [1.29 – 2.27]) were linked to a higher risk of developing MUD. In addition, physical neglect (OR = 1.29 [1.02 – 1.65]), emotional neglect (OR = 1.47 [1.13 – 1.90]), and sexual abuse (OR = 1.46 [1.05 – 2.03]) were associated with an increased risk of MAP. The total number of ACEs also increased the risk of both MUD (OR = 1.28 [1.17 – 1.40]) and MAP (OR = 1.14 [1.05 – 1.24]).

**Conclusions:**

The study demonstrates that MA use patterns and adverse childhood experiences significantly impact the risk of developing MA use disorder and MA-induced psychosis.

**Disclosure of Interest:**

None Declared

